# High Oxygen Evolution Activity of Tungsten Bronze Oxides Boosted by Anchoring of Co^2+^ at Nb^5+^ Sites Accompanied by Substantial Oxygen Vacancy

**DOI:** 10.1002/advs.202002242

**Published:** 2020-09-29

**Authors:** Xiaoning Li, Huan Liu, Yanhua Sun, Liuyang Zhu, Xiaofeng Yin, Shujie Sun, Zhengping Fu, Yalin Lu, Xiaolin Wang, Zhenxiang Cheng

**Affiliations:** ^1^ Institute for Superconducting & Electronic Materials (ISEM) Australia Institute for Innovative Materials Innovation Campus University of Wollongong Squires Way North Wollongong NSW 2500 Australia; ^2^ Department of Materials Science and Engineering University of Science and Technology of China Hefei 230026 P. R. China; ^3^ Henan Collaborative Innovation Center of Energy‐Saving Building Materials Xinyang Normal University Xinyang 464000 P. R. China

**Keywords:** oxygen intercalation mechanisms, oxygen evolution reaction, oxygen vacancies, spin configuration, tungsten bronze

## Abstract

The participation of lattice oxygen in the oxygen evolution reaction (OER) process has been proved to be faster in kinetics than the mechanisms where only metal is involved, although activating the lattice oxygen in the traditional rigid structures remains a big challenge. In this work, efforts are devoted to exploring a new flexible structure that is competent in providing large amounts of oxygen vacancies as well as offering the freedom to manipulate the electronic structure of metal cations. This is demonstrated by anchoring low valence state Co at high valence state Nb sites in the tetragonal tungsten bronze (TTB)‐structured Sr_0.5_Ba_0.5_Nb_2‐_ *_x_*Co*_x_*O_6‐*δ*_, with different ratios of Co to Nb to optimize the Co substitution proportion. It is found that the occupation of Co in the Nb^5+^ sites gives rise to the generation of massive surface oxygen vacancies (O_vac_), while Co itself is stabilized in Co^2+^ by adjacent O_vac_. The coexistence of O_vac_ and LS Co^2+^ enables an oxygen intercalation mechanism in the optimal SBNC45 with specific activity at 1.7 V versus reversible hydrogen electrode that is 20 times higher than for the commercial IrO_2_. This work illuminates an entirely new avenue to rationally design OER electrocatalysts with ultrafast kinetics.

## Graphical abstract

A cobalt‐doped tetragonal tungsten bronze structure Sr_0.5_Ba_0.5_Nb_2‐_ *_x_*Co*_x_*O_6‐*δ*_ is developed as a new oxygen evolution reaction electrocatalyst. This structure is competent in providing large amount of oxygen vacancies as well as the freedom to manipulate the electronic structure. The current density normalized by BET surface area of the optimal sample is 20 times higher than the commercial IrO_2_.

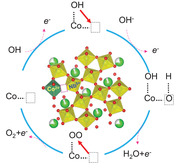

## 1 Introduction

The efficiencies of many green energy technologies are generally restricted by the sluggish kinetics of the oxygen evolution reaction (OER).^[^ ^1^, ^2^, ^3^ ^]^ The underlying mechanisms are gradually being revealed, but they are not fully explicated as yet. For the traditional adsorbate evolution mechanism (AEM),^[^ ^4^, ^5^ ^]^ the rate‐determining step is the O—O bond formation through nucleophilic attack of *O by water or OH^−^ occurring on single metal active sites (Eley–Rideal, ER‐type mechanism).^[^ ^6^, ^7^ ^]^ This step can be avoided by the direct formation of the O—O bond between the neighboring oxygen species on two adjacent active sites (Langmuir–Hinshelwood, LH‐type mechanism).^[^ ^8^ ^]^ In principle, the LH‐type mechanism with two conjunct active sites is more desirable, but it requires the distance between two active sites to be short enough.^[^ ^9^ ^]^ Activating the LH‐type mechanism remains a challenge when only metal cations are OER‐active, as two metal cations are spatially separated by the oxygen anion in oxide catalysts, while the pure transition metals with short metal atom distances are usually unstable as anode.

Recently, O—O formation via direct coupling between a metal cation and a lattice oxygen anion, known as lattice oxygen mechanism (LOM), has been proposed both theoretically and experimentally.^[^ ^10^, ^11^, ^12^ ^]^ In this mechanism, both metal cations and lattice oxygen anions are active sites, cooperating with each other due to the short distance between them. The underlying physics of the LOM is that the electrons involved are contributed by the metal 3d orbitals as well as the O 2p orbitals when Fermi level enters into the O 2p band.^[^ ^13^ ^]^ Exploring new‐type LOM‐active electrocatalysts or turning traditional AEM electrocatalysts into LOM ones by regulation are two common strategies.^[^ ^14^, ^15^, ^16^ ^]^ For instance, CaCoO_3_ is a new LOM high‐efficiency OER electrocatalyst with a significantly small lattice parameter due to the high valence state of Co.^[^ ^17^ ^]^ Unfortunately, this kind of rigid structure is rather rare under ambient conditions, since they require rigorous synthesis conditions such as high temperature and ultrahigh pressure. To turn AEM to LOM in ABO_3_ perovskites, one of the indirect methods applied is A‐site doping to increase the valence state on the B‐site metal active sites, for example, by doping Sr into La sites in the perovskite LaCoO_3‐*δ*_.^[^ ^18^ ^]^ This strategy is usually limited, however, by the crystal lattice tolerance and poor stability issues when the O p‐band center is too close to the Fermi level.^[^ ^19^ ^]^

Compared to the rutile, perovskite, spinel, or pyrochlore structures that are usually adopted by transition metal oxides, the tetragonal tungsten bronze (TTB) structure, represented among a large family of solid solutions with enormous structural flexibility, has not been reported as an OER‐active matrix, although it has been extensively studied in the realms of electro‐optic, piezoelectric, pyroelectric, millimeter wave, and photorefractive applications.^[^ ^20^, ^21^ ^]^ With the typical formula of (A_1_)_4_(A_2_)_2_C_4_B_10_O_30_, the A_1_ cations are in the 15‐fold‐coordinated sites, the A_2_ cations are in the 12‐fold‐coordinated sites, the B cations are in two different sixfold‐coordinated sites, and the C cations are in the ninefold‐coordinated sites.^[^ ^22^ ^]^ Typically, A_1_, A_2_, and C sites can be empty or filled by alkali and alkaline‐earth metal ions, which make the structure relatively flexible and stable. In addition, B sites are octahedrally coordinated, and they are reserved for high valence state cations such as Nb^5+^ or Ta^5+^. What is more interesting, the connections of the NbO_6_ octahedra are versatile in the TTB structure with the Nb—O—Nb distance ranging from 3.71 to 3.94 Å, in contrast to the rigid perovskite structure, for example, LaCoO_3_ (Co—O—Co 3.82 Å) and SrCoO_3_ (Co—O—Co 3.86 Å). Thus, we consider that the flexible TTB structure could be an excellent matrix, capable of fast OER mechanisms, if it can accommodate enough OER‐active sites.

In this work, we attempted to anchor Co on Nb^5+^ sites in Sr_0.5_Ba_0.5_Nb_2_O_6_ (SBN), to explore the possible OER activity and mechanisms in the TTB structure. The similarity in the ion radii of Co and Nb ensures the success of substitution, while the difference in valence could introduce a huge amount of oxygen defects (Co^2+^ (LS, 0.79 Å) vs Nb^5+^ (0.78 Å)), with the great potential of activating faster mechanisms with the involvement of oxygen defects. Sr_0.5_Ba_0.5_Nb_2‐_ *_x_*Co*_x_*O_6‐*δ*_ (*x* = 0, 0.3, 0.45, 0.6, denoted as SBN, SBNC30, SBNC45, and SBNC60, respectively) with different amounts of Co was prepared to optimize the proportion of substitution. The crystal structure results suggest that the TTB structure can be well maintained in the SBN, SBNC30, and SBNC45, but the perovskite secondary phase would emerge in the SBNC60. Electronic study suggests that the occupation of Co in the Nb^5+^ sites of TTB structure brings about massive oxygen vacancy (O_vac_) and stabilizes itself in the form of Co^2+^. The presence of O_vac_ leads to a spin state transition of Co^2+^ from high spin (HS) to low spin (LS) due to the further crystal field splitting. The coexistence of O_vac_ and LS Co^2+^ triggers an oxygen intercalation mechanism, endowing SBNC45 with superior OER performance to commercial IrO_2_ under alkaline working conditions.

## 2 Results and Discussion

### 2.1 Crystal and Electronic Structure

**Figure 1:**
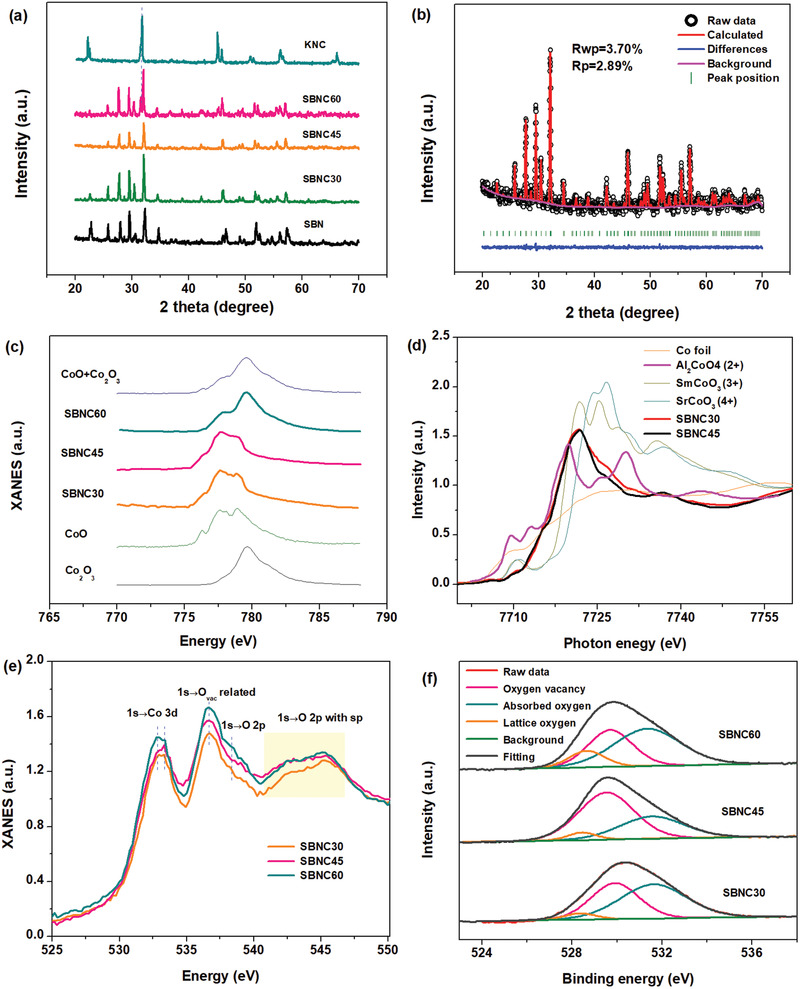
Structure characterization spectra. a) XRD patterns of SBN, SBNC30, SBNC45, SBNC60, and KNC; b) refined XRD pattern of SBNC45 as a demonstration; c) Co L_3_^−^ edge XAS spectra recorded on the as‐prepared samples as well as standard references; d) Co K‐edge XAS spectra of SBNC30 and SBNC45, and plots downloaded from https://materialsproject.org/ for comparison; e) O K‐edge XAS spectra of SBNC30, SBNC45, and SBNC60; f) fitted XPS core level spectra of O 1s from SBNC30, SBNC45, and SBNC60.

From the X‐ray powder diffraction patterns (XRD) patterns in **Figure** **1**a, we can see that the original TTB structure can be maintained when *x* ≤ 0.45. A secondary phase identical to perovskite KNbO_3_ (cubic, space group *Pm‐*3*m*) would emerge, however, when *x* ≥ 0.6. Thus, a Co‐doped KNbO_3_ perovskite with same Co/Nb ratio (KNC) was also prepared for a better comparison, the XRD pattern of which is also provided in Figure 1a. The specific crystal structure information can be obtained by the Rietveld refinement method, based on TTB Sr_0.5_Ba_0.5_Nb_2_O_6‐*δ*_ (tetragonal, space group *P*4*bm*) prototype. The refined pattern of SBNC45 is displayed in Figure 1b (for other samples, see Figure S1, Supporting Information). The profile and weighted profile *R* factors (*R*_p_ and *R*_wp_) are far smaller than 7% for the SBN, SBNC30, and SBNC45 samples, indicating the pure TTB structure phase when *x* ≤ 0.45. The refined lattice parameters are listed in **Table** **1** (for more detailed crystallographic information, see Table S1, Supporting Information).

**Table 1:** Lattice parameters from XRD refinement

Parameter	SBN	SBNC30	SBNC45
*a*	12.48 Å	12.50 Å	12.52 Å
*b*	12.48 Å	12.50 Å	12.52 Å
*c*	3.96 Å	3.95 Å	3.95 Å
*V*	612.1 Å^3^	617.2 Å^3^	619.2 Å^3^

The enlargement of the unit cell from SBN to SBNC45 can be clearly observed from Table 1, implying that Co has been successfully doped into the TTB crystal lattice. If Co cations are not incorporated into the TTB lattice, the unit cell is likely to shrink with reduced lattice parameters, given the insufficient supply of both Nb and Co. On the basis of the radii of the cations listed in **Table** **2**, Co ions should have to occupy Nb sites in the TTB lattice. In addition, from original designs, only Nb sites with suitable size (0.78 Å) are left as vacancies reserved for Co, and Ba or Sr sites are well allocated with large cations (at least larger than 1.32 Å). On the contrary, if Co cations are located at Ba or Sr sites, then the Ba or Sr should have to find sites that are large enough for them, which is not available in the TTB structure. What is noteworthy is that there is a large probability that Co^2+^ rather than Co^3+^ enters Nb sites because the smaller radius of Co^3+^ (irrespective of the spin state) might also lead to lattice shrinkage, which is opposite to the XRD results. Herein, we draw a preliminary conclusion that Co has been successfully anchored in Nb sites with the valence state of Co^2+^.

**Table 2:** Parameters of cations in different coordination environments

Cations	Electronic	Coordination	Spin state	Crystal radii^a)^	t_2g_ Electron	*e*_g_ Electron
Co^4+^	3d^5^	6	High spin	0.67 Å	3	2
Co^3+^	3d^6^	6	Low spin	0.69 Å	6	0
Co^3+^	3d^6^	6	High spin	0.75 Å	4	2
Co^2+^	3d^7^	6	Low spin	0.79 Å	6	1
Co^2+^	3d^7^	6	High spin	0.89 Å	5	2
Nb^5+^	4d^0^	6	–	0.78 Å	–	–
Sr^2+^	5s^0^	6	–	1.32 Å	–	–
Sr^2+^	5s^0^	12	–	1.58 Å	–	–
Ba^2+^	6s^0^	6	–	1.49 Å	–	–
Ba^2+^	6s^0^	12	–	1.75 Å	–	–

a) Data are from http://abulafia.mt.ic.ac.uk/shannon/ptable.php.

To verify the above conclusion, XAS spectra of the Co L_3_‐edge were first conducted as shown in Figure 1c. Spectra of SBNC30 and SBNC45 coincide with the standard CoO (Co^2+^), while SBNC60 sample contains both Co^2+^ and Co^3+^ compared to the spectra of reference samples CoO (Co^2+^) and Co_2_O_3_ (Co^3+^). This implies that the TTB structure has the capacity to stabilize Co^2+^ cations in its lattice, while SBNC45 encompasses the maximum amount of Co^2+^. Doping excessive Co into SBNC60 would generate a perovskite secondary phase containing Co^3+^. This result can also be confirmed by the Co K‐edge X‐ray absorption near‐edge structure (XANES) spectra in Figure 1d, which reflect the electronic dipole transition from the 1s level to the 4p levels. The absorption edge position of SBNC30 and SBNC45 agrees with the Co^2+^ from the standard Al_2_CoO_4_. The pre‐edge peak at 7100 eV is visible in tetrahedral coordination, originating from the electron transition from the Co 1s to 3d orbitals. The octahedrally coordinated SBNC30 and SBNC45 reflect a certain degree of lattice distortion of the CoO_6_ octahedra from the central symmetry.^[^ ^23^ ^]^

The specific spin structure of Co^2+^ can be estimated based on the temperature dependence of the magnetization (*M*−*T*) curve and the Curie–Weiss law fitting.^[^ ^24^, ^25^ ^]^ From Figure S2, Supporting Information, we can see that the magnetization of all samples decreased with increasing temperature, and the inverse magnetic susceptibility curves fit very well with the Curie–Weiss law, suggesting an overall paramagnetic ground state. After calculation, the number of unpaired electrons of Co^2+^ in SBNC30 is found to be near 3, but it decreased to about 2 in SBNC45 (details in Table S2, Supporting Information). This result implies that SBNC45 encompasses a larger proportion of LS Co^2+^ with fewer unpaired electrons, while SBNC30 contains more HS Co^2+^ with more unpaired electrons. It should be noted that the spin configuration estimated based on Curie–Weiss law is the average information for the bulk.

The original Nb sites are in the 5+ valance state in the TTB structure, but they accommodate Co^2+^, which is reminiscent of a large amount of O_vac_ generation reducing the valence state of Co for the principle of electrical neutrality during the crystal growth. The generation of O_vac_ can be confirmed by the O K‐edge XAS spectra in Figure 1e. Since only electron transition from O 1s to unoccupied states with O 2p character can be probed in O K‐edge XAS, it gives information on the density of unoccupied O 2p states and hybridizing with transition metal 3d states.^[^ ^26^ ^]^ The peak at 538.4 eV represents the transition from O 1s to O 2p and the broad peak range of 540–545 eV contains the transitions to O 2p hybrids with metal sp orbitals.^[^ ^27^ ^]^ The absorption around 532.8–533.4 eV is caused by the electron transition from O 1s to Co 3d, while the absorption peak located at 536.7 eV reflects the electron transition from O 1s to O_vac_ related unoccupied 2p orbitals.^[^ ^28^ ^]^ O K‐edge XAS spectra with the features of the transition from O 1s to O_vac_ related unoccupied 2p orbitals confirm the existence of O_vac_ on the surface.

Further solid evidence can be observed in the O 1s core‐level spectra in Figure 1f. The peaks with binding energy (BE) around 528.3–528.8 eV are signals from the electron transition of lattice oxygen (O_L_). The peak ranging 531.6–531.7 eV is most likely contributed by surface adsorbed oxygen (O_ad_), while the peak centered at 529.6–529.9 eV can be assigned to the less electron‐rich oxygen species related to the oxygen vacancies (O_vac_).^[^ ^29^ ^]^ As summarized in **Table** **3**, the peak area from O_vac_ related species is substantial for all the samples, from 41.1% for SBNC30 and 61.4% for SBNC45 to 39.7% for SBNC60, indicating that enormous amounts of O_vac_ are generated on their surfaces. Raman spectra investigated at room temperature in the frequency range of 100–1100 cm^‐1^ can also provide some structural information (Figure S3, Supporting Information). The spectrum of SBN obtained here is well consistent with previous reports.^[^ ^30^, ^31^ ^]^ Spectra of SBNC30 and SBNC45 are similar to that of SBN but with obvious peak broadening, which is caused by additional disorder introduced by O_vac_ with cobalt substitution. Due to the complexity of the TTB structure, there are 45 atoms and 135 vibrational modes in the unit cell. Remarkably, there is the strongest peak located at around 834 cm^‐1^ in SBNC30, SBNC45, and SBNC60, which cannot be observed in the SBN. This peak is identified as a vibrational mode of the CoO_6_ octahedron, as detected in cobalt‐doped layered perovskite oxides.^[^ ^32^ ^]^ Therefore, the emerging of this vibrational mode of the CoO_6_ octahedron confirms that Co cations are octahedrally coordinated, excluding the possibility that Co may be located in the Ba/Sr sites, which are not octahedral environments.

**Table 3:** Fitting parameters of O 1s core‐level spectra

Sample	Peak 1 [eV]	Ratio	Peak 2 [eV]	Ratio	Peak 3 [eV]	Ratio
SBNC30	528.3	4.8%	529.9	41.1%	531.6	33.9%
SBNC45	528.5	4.7%	529.6	61.4%	531.7	54.1%
SBNC60	528.8	6.9%	529.7	39.7%	531.5	53.7%

**Figure 2:**
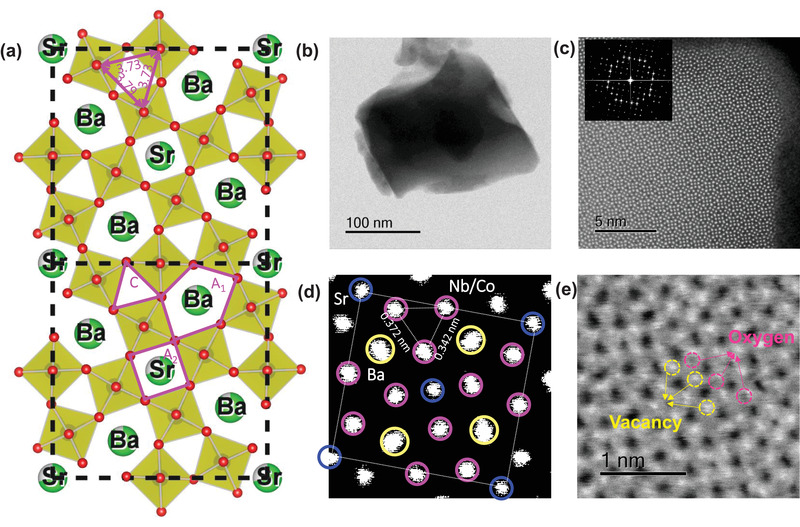
a) Two unit cells projected on the (001) plane, based on the refined structure of SBNC45; all A sites are occupied by metal cations, but, in fact, they are partially occupied but randomly distributed; b) TEM image of a SBNC45 particle around 200 nm in size; c) HAADF image from the edge of the particle in (b), and the inset is the corresponding SAED pattern by fast Fourier transform (FFT); d) enlarged HAADF image from (c) with the superposition of one unit cell marked with circles and lines; e) ABF image with high magnification, in which the dark area marked in red circles is the oxygen atom column, while the bright area marked in yellow circles denotes the oxygen vacancies.

**Figure** **2**a presents the calculated crystal structure of SBNC45 based on the XRD refinement with two cells projected in the (001) plane. On average, the distances between Nb/CoO_6_ octahedra are 3.73 (50%), 3.79 (25%), and 3.97 Å (25%). The high percentage with the short distance of 3.73 Å makes the average distance between Nb/Co around 3.81 Å, which is smaller than for other Co based perovskite oxides such as LaCoO_3_ (3.82 Å) and SrCoO_3_ (3.86 Å) (Figure S4, Supporting Information). Figure 2b is a transmission electron microscope (TEM) image of a single crystal SBNC45 particle around 200 nm in size. It is noteworthy that some of the particles are polycrystals with a broad size distribution due to the high sintering temperature. The average particle size is about 1.5 µm, estimated from the scanning electron microscopy (SEM) image (Figure S5, Supporting Information), so the sample has a relatively small BET surface area (≈2.6 m^2^ g^−1^, Figure S6, Supporting Information). The SEM‐energy dispersive spectroscopy (EDS) elemental mapping in Figure S7, Supporting Information, suggests that the Sr, Ba, Nb, and Co elements are distributed homogeneously throughout the particles.

The TEM image in Figure 2c is a high‐angle annular dark‐field scanning transmission electron microscopy (HAADF‐STEM) image recorded with the electron beam along the [001] zone axis, from which we can see that these atoms are highly organized in a crystalline pattern. The inset is the selected area electron diffraction (SAED) pattern corresponding to the lattice from the HAADF image, indicating a good single crystallinity in that area. The enlarged HAADF image in Figure 2d with the superposition of a unit cell of the TTB structure confirms the pure phase of the as‐prepared samples despite the Co doping. There are two adjacent Co/Nb sites as noted, with a spacing of 3.72 Å, which is consistent with the value of 3.73 Å from the XRD refinement. A distance of 3.42 Å for the other two adjacent Co/Nb cations is also observed, however, which is a characteristic of lattice distortion induced by O_vac_. Figure 2e is an annular bright‐field scanning transmission electron microscope (ABF‐STEM) image, enabling us to observe the atomic columns of both relatively heavy elements (metals) and light elements (oxygen) with the same contrast. The electrons that are incident on the O_vac_ columns will pass through directly, appearing as bright dots marked by yellow circles in the image. If oxygen atoms are present in the crystal lattice, then black dots will be observed instead, as marked with pink circles. It is obvious that the bright dots account for a dramatically larger proportion than the black dots in Figure 2e. This result from ABF is another piece of solid evidence that strongly affirms the existence of abundant oxygen vacancies (O_vac_) on the surfaces of the as‐prepared samples.

### 2.2 Electrochemical OER Performance

**Figure 3:**
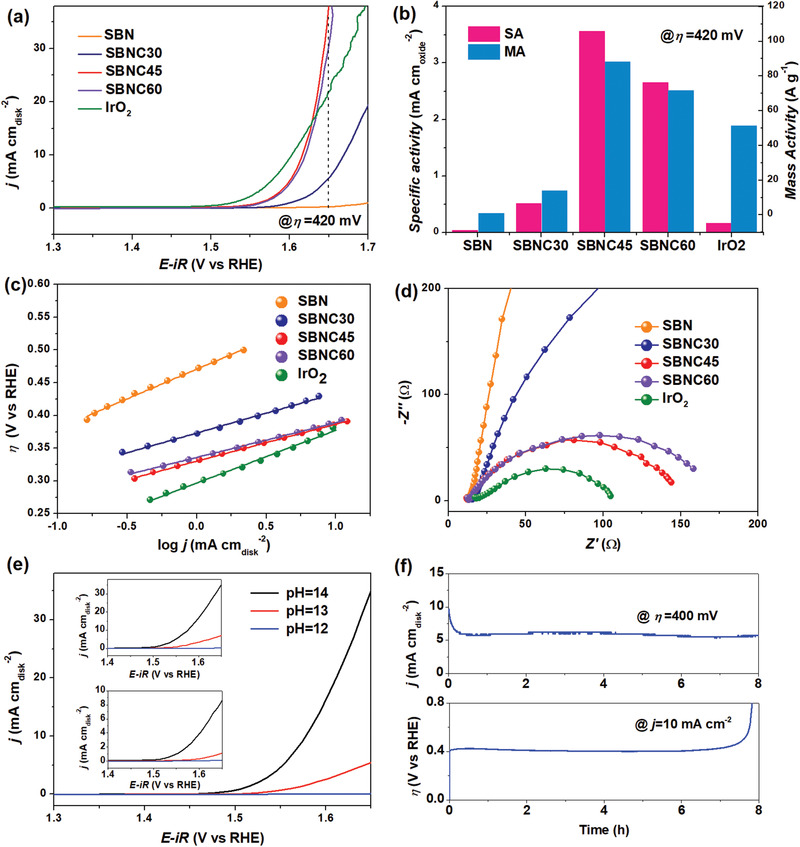
a) LSV curves collected at the scan rate of 5 mV s^−1^ in 1 M NaOH; b) SA and MA at the overpotential of 420 mV; c) Tafel plots with the scan rate of 5 mV s^−1^; d) Nyquist plots measured at 1.6 V versus RHE, ranging from 1 to 10^5^ Hz; e) LSV curves of SBNC45 at the scan rate of 5 mV s^−1^ in 0.01 M NaOH, 0.1 M NaOH, and 1 M NaOH, insets are the corresponding curves measured on SBNC30 (top) and SBNC60 (bottom); f) CA curve (top) at the overpotential of 400 mV and CP curve (bottom) at the current density of 10 mA cm^−2^ with the duration of 8 h tested on the SBNC45.

Linear sweep voltammetry (LSV) curves tested in 1 M NaOH electrolyte are displayed in **Figure** **3**a, in which the current density (*j*) is calibrated by the disk electrode area (0.07065 cm^2^). The current density at 1.7 V versus RHE is 1.0, 12.9, 30.9, and 29.2 mA cm^−2^ for SBN, SBNC30, SBNC45, and SBNC60, respectively. The overpotential (*η*) at 10 mA cm^−2^ is about 460, 400, and 401 mV for the SBNC30, SBNC45, and SBNC60 samples, respectively. As expected, SBNC45 performed better than SBNC30 because more Co active sites were involved, although SBNC60 with more cobalt content exhibits similar OER activity to that of SBNC45. This phenomenon can be well explained by the crystal and electronic result that excessive Co in SBNC60 forms a perovskite secondary phase, as Co^3+^ in the perovskite secondary phase might not as active as Co^2+^ in the TTB structure. This is evidenced by the negligible OER performance of the perovskite KNC with the same Co ratio prepared by the same method (Figure S8, Supporting Information). Thus, when part of the surface of TTB is covered by perovskite, it would result in a slightly reduced OER performance. The values of Tafel slopes can be calculated based on the Tafel curves in Figure 3c: 91, 60, 57, and 53 mV dec^−1^ for SBN, SBNC30, SBNC45, and SBNC60, respectively. Even though the Tafel slope of SBNC60 is slightly smaller than that of SBNC45, SBNC45 has a lower overpotential at log*j* = 0. Thus, the result from the Tafel slope is in good agreement with that obtained from the LSV curves. To provide a clear comparison between these electrocatalysts, the mass activity (MA) and specific activity (SA) at overpotential *η* = 420 mV for all the samples are displayed in Figure 3b. The MA is the current density based on the current normalized by the corresponding mass of electrocatalyst, which is more useful in practical applications. The SA normalized by the total surface area can reflect the intrinsic activity of the catalyst, which is significant for fundamental research. In Figure 3b, an upward tendency can be observed from SBN to SBNC45, and then a downward tendency when it comes to SBNC60. This general dependency is even more distinctive when evaluated from the SA rather than from the MA, which indicates that the intrinsic OER activity is even more different. We note that SBNC45, SBNC60, and even SBNC30 are better than state‐of‐the‐art IrO_2_ in terms of their intrinsic activity according to the SA results.

The Nyquist plots in Figure 3d show that the polarization resistance (semicircle diameter) is decreased dramatically from SBN to SBNC60, which means that the electron transfer barrier is greatly reduced with increasing Co content. Being proportional to the electrochemical surface area (ECSA), the double‐layer capacitance (*C*_dl_) can provide another comparison. The calculated *C*_dl_ derived from the cyclic voltammograms (CVs, Figure S9, Supporting Information) versus the scan rate is 1.10 mF cm^−2^ on SBNC30, 4.02 mF cm^−2^ on SBNC45, and 1.03 mF cm^−2^ on SBNC60. The higher *C*_dl_ value of SBNC45 implies that more enriched active sites are involved. Furthermore, LSV measurements on SBNC30 and SBNC45 were also conducted in NaOH electrolytes with different pH values. As shown in Figure 3e and the insets, the OER activities of SBNC30, SBNC45, and SBNC60 all exhibit a strong pH‐dependent characteristic, implying a non‐concerted proton–electron transfer related OER mechanism.^[^ ^33^ ^]^ Long‐term activity and stability were tested on SBNC45, as shown in Figure 3f. Both the chronoamperometry (CA) at the overpotential of 400 mV and the chronopotentiometry (CP) at the current density of 10 mA cm^−2^ were stable over a duration of 8 h, confirming the good performance stability of the SBNC45 sample. With respect to its structural stability, the high‐resolution TEM (HRTEM) in Figure S10, Supporting Information, suggests that SBNC45 can maintain its TTB structure with no obvious structural deterioration or surface amorphization.

### 2.3 Discussion

**Figure 4:**
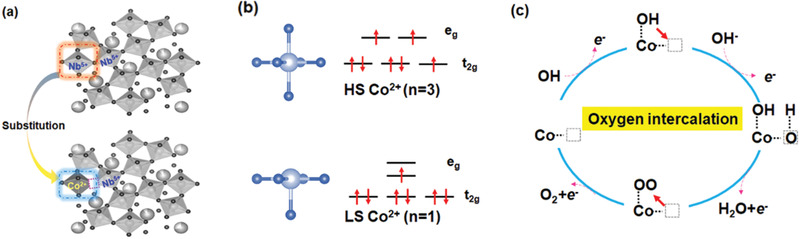
a) Demonstration of the replacement of Nb^5+^ by Co in the TTB structure; b) spin configuration evolution from HS Co^2+^ to LS Co^2+^ in the octahedron due to further crystal field splitting caused by the generation of O_vac_; c) a possible oxygen intercalation mechanism, with the dashed box representing the O_vac_.

The greatly improved OER performance of the cobalt doped TTB structured samples is likely to have originated from the allocation of low valence state Co^2+^ to the original high valence state Nb^5+^ sites, which leads to the generation of a tremendous amount O_vac_ (**Figure** **4**a). One of the most amazing merits of the TTB structure is its unique octahedral connection network that is flexible enough to accommodate Co^2+^ cations together with O_vac_. On the one hand, O_vac_ on the surface facilitates the adsorption of OH^−^ in the interface, as experimentally and theoretically proved by many researchers.^[^ ^13^, ^18^ ^]^ On the other hand, one O_vac_ in an oxygen containing octahedron could transform the coordination to pyramidal, with a degeneration of the 3d orbital energy, as illustrated in the diagram in Figure 4b. The evolution of this electronic structure will lead to a decrease in the number of unpaired electrons, which is confirmed by the results of the magnetization characterization.

SBNC45, despite its poor electronic conductivity, exhibits 20 times higher current density than IrO_2_ at 1.7 V versus RHE when normalized by the BET surface area. It also has a rather small Tafel slope value (<70 mV dec^−1^) together with a pH‐dependent characteristic, which indicates ultrafast non‐concerted proton–electron transfer during the OER. This should be promoted by the combination of Co^2+^ and O_vac_ which are connected as Co—O_vac_—Co when the Co sites are adequate. In this situation, an O_vac_‐mediated oxygen intercalation mechanism may be possible, similar to the oxygen intercalation in the LaMnO_3_ perovskite pseudocapacitor or the O_vac_ filling mechanism in CuO/CeO_2_ catalysts.^[^ ^34^, ^35^ ^]^ As demonstrated in Figure 4c, starting from the adsorption of OH^−^ by a Co site on the surface in alkaline OER conditions, oxygen from OH· may be intercalated into the lattice when an adjacent lattice oxygen is absent (O_vac_ site). Compared to HS Co^2+^, LS Co^2+^ with less unpaired electrons and more empty orbitals is more efficient in the adsorption of OH^−^. More O_vac_ makes oxygen intercalation easier. What is more important, the oxidation state of Co is increased after oxygen intercalation, which is more OER‐active. Figure S11, Supporting Information, is the Co L_3_^−^edge XAS spectra recorded on SBNC45 samples before and after the OER tests, from which it is observed that the Co cations on the surface have an electronic structure evolution from Co^2+^ to higher oxidation states.

According to the Zaanen–Sawatzky–Allen (ZSA) model, the electronic structure of transition metal oxides is typically described by three primary parameters: the Hubbard interaction (self‐exchange), the metal–oxygen covalency (charge‐transfer energy), and the metal–oxygen hybridization (orbital overlapping).^[^ ^36^ ^]^ The oxygen intercalation in the O_vac_ introduces high covalency between Co and oxygen, which will reduce the charge‐transfer energy due to the increased ionization energy of Co cations with high valence. A small charge‐transfer energy can alter the OER mechanism from concerted proton–electron transfer to non‐concerted proton–electron transfer reactions. For example, in the concerted proton–electron transfer process, the addition of OH^−^ and the loss of *e^−^* from O* to OOH* are coupled, which is pH‐independent. They will be separated, however, in the non‐concerted proton–electron transfer with a pH‐dependent characteristic. A typical non‐concerted proton–electron transfer mechanism is the LOM, in which O‐O would directly be formed through bonding of O* on a metal active site with lattice oxygen. However, part of the oxygen in O_2_ is from the crystal lattice for the traditional LOM electrocatalysts, which will lead to structural stability issues such as amorphization. In this oxygen intercalation mediated mechanism, the entire oxygen element is from the electrolyte, even though some oxygen has even been inserted into the crystal lattice. Therefore, the electrocatalyst can maintain its original structure before and after OER tests.

We believe that the OER‐active metal cations, enough O_vac_, and a flexible structure are the essential conditions for this oxygen intercalation mechanism. It was evidenced by several reference samples prepared and tested under the same conditions (Figures S12–16, Supporting Information). For example, the SBNF45 sample with the same amount of Fe doping into the Nb^5+^ sites, is not OER active, possibly due to the inefficient metal cations (Fe^3+^), while the (SBC)N sample with Co doping into Sr^2+^ or Ba^2+^ sites is also not OER‐active without adequate O_vac_. Similarly, bad OER performances were also observed from the LNC075 and LNC225 samples with the same amount of Co occupying the Nb^5+^ sites in the trigonal crystal structure. The OER performance is limited by the rigid crystal structure, as in the situation of the perovskite KNC.

## 3 Conclusion

High intrinsic OER activity is reported in the Co‐doped TTB structure for the first time. TTB can maintain its structure with the harmonious coexistence of low‐valence Co^2+^ and a huge amount of O_vac_. Due to this magic combination, the as‐prepared SBNC45 sample exhibits superior performance to the state‐of‐the‐art IrO_2_ with a very small Tafel slope and a pH‐dependent characteristic. The oxygen intercalation‐mediated mechanism can expedite the OER process by non‐concerted proton‐electron transfer, but without compromising of the structure and the performance stability. This work indicates that the oxygen intercalation mechanism with a high reaction rate and good stability can be instructive for designing new electrocatalysts and that doping low valence state cations into high valence sites in the flexible structure is an effective strategy.

## 4 Experimental Section

##### Synthesis

All the samples in the present work were prepared by conventional solid‐state reaction (SSR). Nb_2_O_5_ (99.99%), Ba(NO_3_)_2_ (≥99%), Sr(NO_3_)_2_ (≥99%), Co(NO_3_)_2_·6H_2_O (≥98%), KNO_3_ (≥99%), LiNO_3_ (99.99%), and Fe_2_O_3_ (99.995%) were purchased from Aldrich Ltd. and used as starting materials. For the preparation of the SBN, SBNC30, SBNC45, and SBNC60 samples, the corresponding chemicals were mixed homogeneously in stoichiometric ratios according to the formula Sr_0.5_Ba_0.5_Nb_2‐_ *_x_*Co*_x_*O_6‐*δ*_ (*x* = 0, 0.3, 0.45, 0.6). The mixed powders were first pressed into pellets under axial pressure (8 MPa) and calcinated at 700 °C for 2 h in air. After cooling down, these pellets were reground into fine powders and repressed into pellets under axial pressure (12 MPa). After sintering at 1150 °C for 12 h in air atmosphere, all the pellets were ground for 2 h before characterizations and electrochemistry tests. Sr_0.5_Ba_0.5_Fe_0.45_Nb_1.55_O_6_ denoted as SBNF45 and Sr_0.4_Ba_0.4_Co_0.2_Nb_2_CoO_6_ denoted as (SBC)N were also prepared under the same conditions. KNb_0.775_Co_0.225_O_3_ (KNC), LiNb_0.925_Co_0.075_O_3_ (LNC075), and LiNb_0.775_Co_0.225_O_3_ (LNC225) were prepared by following the same procedure except for their relatively lower final sintering temperature (1000 °C). Commercial IrO_2_ (99.9%) with a Brunauer–Emmett–Teller (BET) surface area of ≈32.5 m^2^ g^−1^ was also purchased from Aldrich Ltd. and tested after grinding.

##### Materials Characterization

XRD were recorded on a GBC eMMA X‐ray diffractometer with Cu‐K*α* radiation and refined by the Pawley method. SEM and element mapping images were obtained on a JEOL JSM‐7500FA and atomic arrangements from HAADF and ABF observations were conducted on a scanning transmission electron microscope (STEM, JEOL JEM‐ARM200F). The BET (Nova 1000, Quantachrome Instrument) surface area was estimated based on the nitrogen adsorption/desorption isotherms. Raman spectra were collected at room temperature with a SPEX‐1403 Laser Raman spectrometer excited by a 532 nm wavelength Ar^+^ laser. Magnetization–temperature curves were measured under a 500 Oe magnetic field within the temperature range of 5‒340 K on a Quantum Design physical properties measurement system (PPMS‐9T) (Quantum Design, USA) with the vibrating sample magnetometer (VSM) option. Electronic structure was analyzed based on X‐ray photoelectron spectra (XPS) collected on a SPECS XPS/Auger spectrometer, Co L‐edge soft X‐ray absorption spectra (XAS) from the BL12B‐a facility of the National Synchrotron Radiation Laboratory (NSRL, P. R. China), O K‐edge spectra from the Soft X‐ray Spectroscopy beamline at the Australian Synchrotron (AS, Australia), and Co K‐edge spectra from BL14W1 facility of the Shanghai Synchrotron Radiation Facility (SSRF, P. R. China).

##### Electrochemical Testing

For the preparation of working electrodes, 0.75 mL deionized water and 0.25 mL isopropanol, 2 mg carbon powder, 10 mg of electrocatalyst, and 0.1 mL of Nafion 117 solution were mixed under ultrasound for 1 h to form a homogeneously mixed ink, and then 3 µL of above ink was drop casted onto a glassy carbon (GC) electrode 3 mm in diameter (effective electrode area 0.07065 cm^2^, mass loading 0.38 mg cm^−2^). OER performances were obtained on an electrochemical workstation (Ivium‐n‐Stat, Ivium Technologies) and a standard three‐electrode electrochemical cell with saturated Ag/AgCl as reference electrode and platinum wire as the counter electrode. All the measurements were carried out in 1 M NaOH aqueous solution except for the pH‐dependent LSV test. All LSV curves and Tafel plots were measured at a scan rate of 5 mV s^−1^. Electrochemical impedance spectroscopy (EIS) was conducted at 1.6 V versus reversible hydrogen electrode (RHE), over frequencies from 1 to 10^5^ Hz. All the potentials versus Ag/AgCl were converted to versus RHE according to the Nernst Equation (1)

(1) $$E_{\mathrm{RHE}}=E_{\mathrm{Ag}/\mathrm{AgCl}}+E_{\mathrm{Ag}/\mathrm{AgCl}}^{0}+0.059\times \mathrm{pH}$$

where *E*_RHE_ is the converted potential versus RHE, $E_{\mathrm{Ag}/\mathrm{AgCl}}^{0}$ = 0.1976 V, and *E*_Ag/AgCl_ is the measured potential against the Ag/AgCl reference. Potentials are *iR*‐corrected by using the current (*i*) and solution resistance value (*R*) resolved from the Nyquist plots.

## Conflict of Interest

The authors declare no conflict of interest.

## Supporting information

Supporting Information
