# Supplementary material for: High Oxygen Evolution Activity of Tungsten Bronze Oxides Boosted by Anchoring of Co2+ at Nb5+ Sites Accompanied by Substantial Oxygen Vacancy
Source: Adv Sci (Weinh). 2020 Sep 29;7(22):2002242. doi: 10.1002/advs.202002242 (PMC7675188; doi:10.1002/advs.202002242)
Supplement: Supplementary file 1 — Supporting Information [file ADVS-7-2002242-s001.pdf]

© 2020 Wiley-VCH GmbH

ADVANCED SCIENCE
Open Access

Supporting Information

for *Adv. Sci.,* DOI: 10.1002/advs.202002242

High oxygen evolution activity of tungsten bronze oxides boosted by anchoring of Co2+ at Nb5+ sites accompanied by substantial oxygen vacancy

Xiaoning Li, Huan Liu, Yanhua Sun, Liuyang Zhu, Xiaofeng Yin, Shujie Sun, Zhengping Fu, Yalin Lu, Xiaolin Wang, and Zhenxiang Cheng**

WILEY-VCH

© 2020 Wiley-VCH GmbH

Supporting Information

High oxygen evolution activity of tungsten bronze oxides boosted by anchoring of Co^{2+} at Nb^{5+} sites accompanied by substantial oxygen vacancy

Xiaoning Li, Huan Liu, Yanhua Sun, Liuyang Zhu, Xiaofeng Yin, Shujie Sun, Zhengping Fu, Yalin Lu, Xiaolin Wang, and Zhenxiang Cheng**

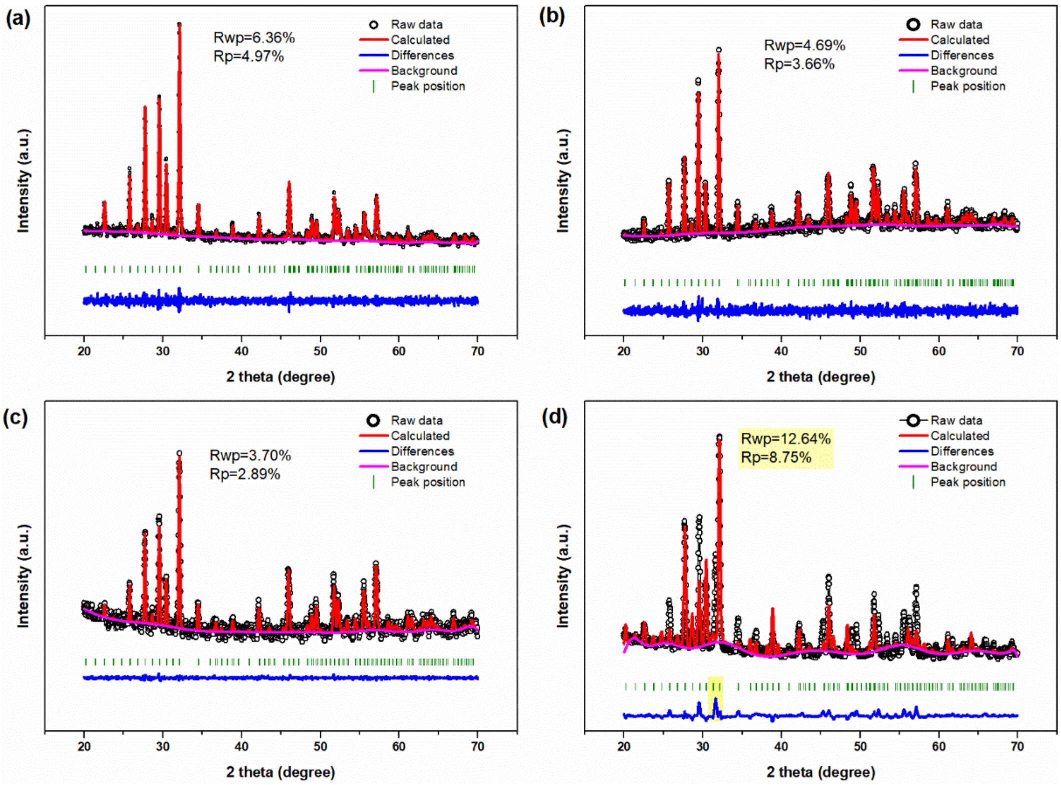

Figure S1. Refined XRD patterns based on the TTB $Sr_{0.5}Ba_{0.5}Nb_2O_{6-\delta}$ (Tetragonal, space group P4bm): (a) SBN; (b) SBNC30; (c) SBNC45, and (d) SBNC60, in which the marked peak in the Differences curve was most probably induced by the perovskite secondary phase

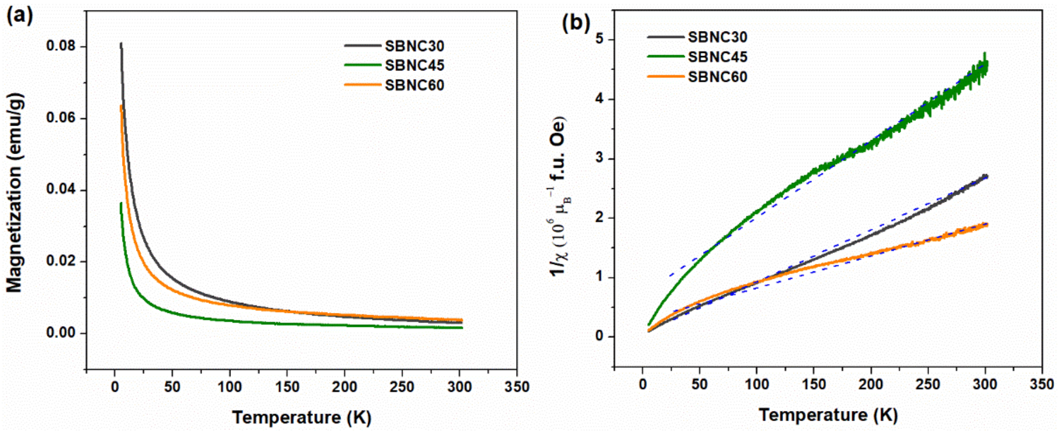

Figure S2. (a) Magnetization–temperature (*M*-*T*) curves for SBNC30, SBNC45, and SBNC60; (b) corresponding curves of inverse magnetic susceptibility versus temperature.

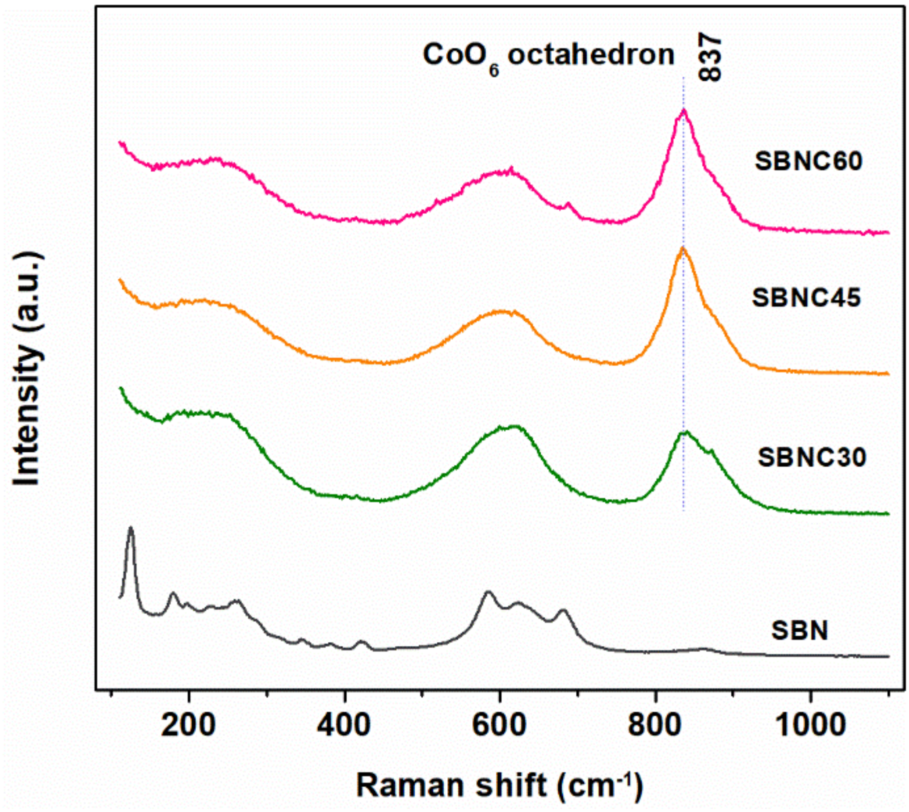

Figure S3. Raman spectra of SBN, SBNC30, SBNC45, and SBNC60 at room temperature. The peak located at 837 cm^{-1} is attributed to the vibration modes of CoO_6 octahedra.

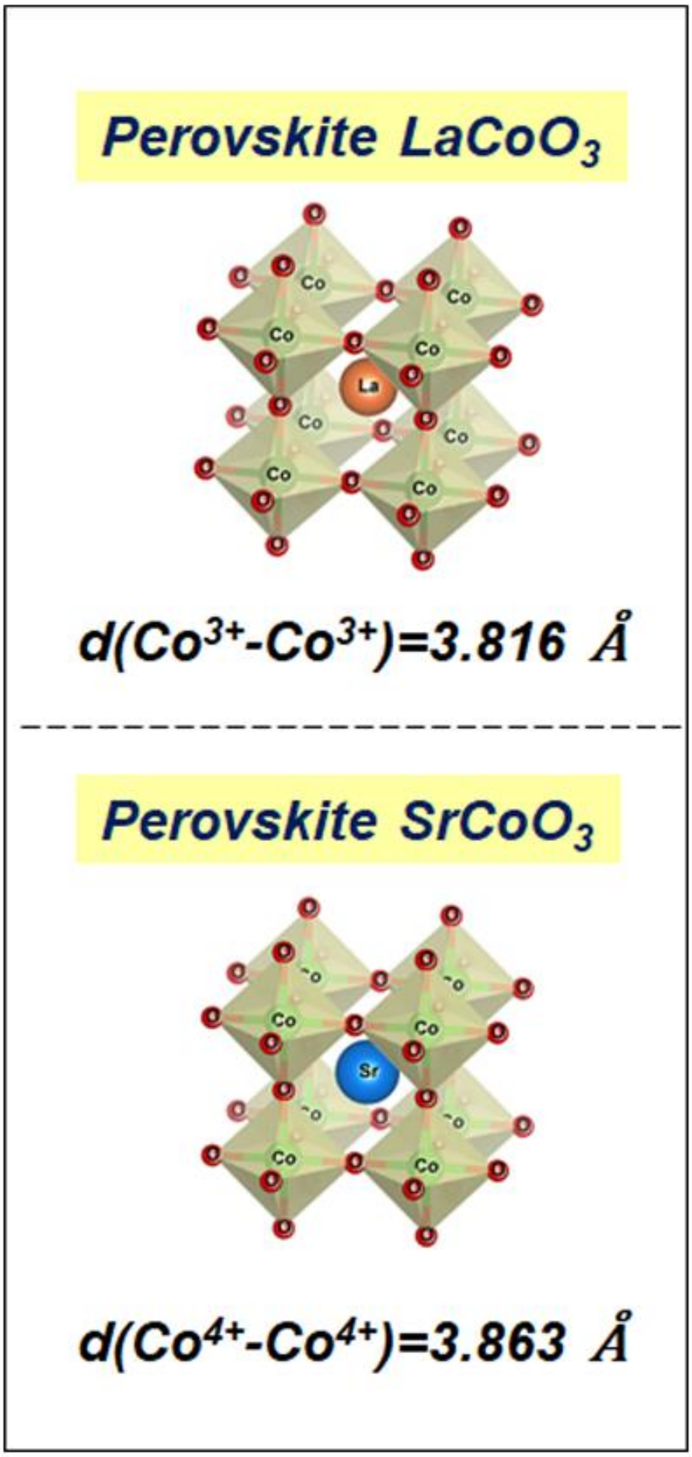

Figure S4. Standard crystal structures of $LaCoO_3$ and $SrCoO_3$

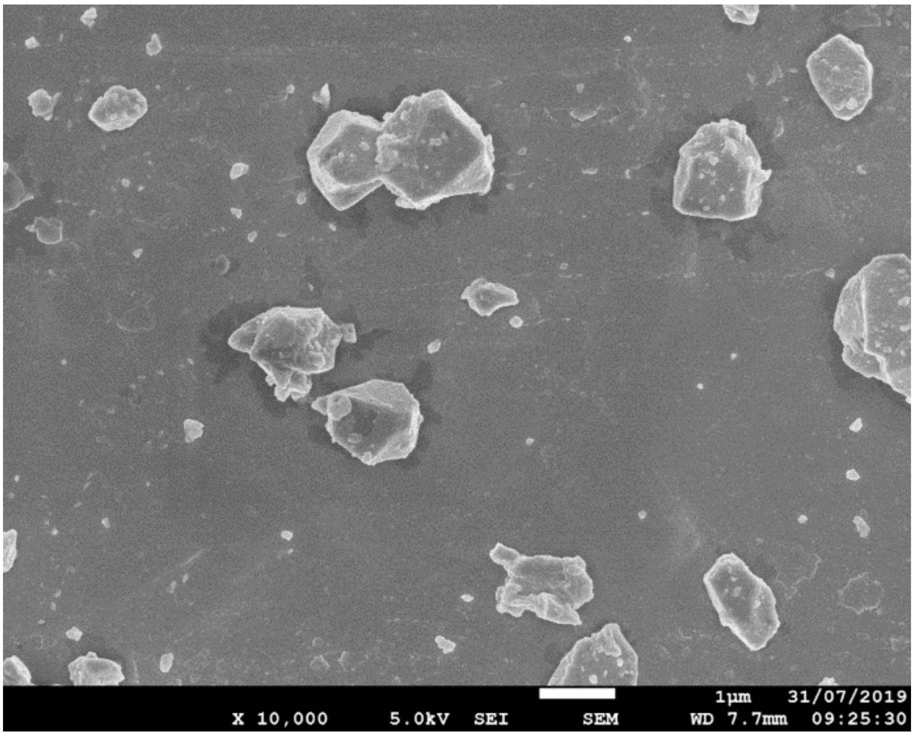

Figure S5. SEM image of SBNC45.

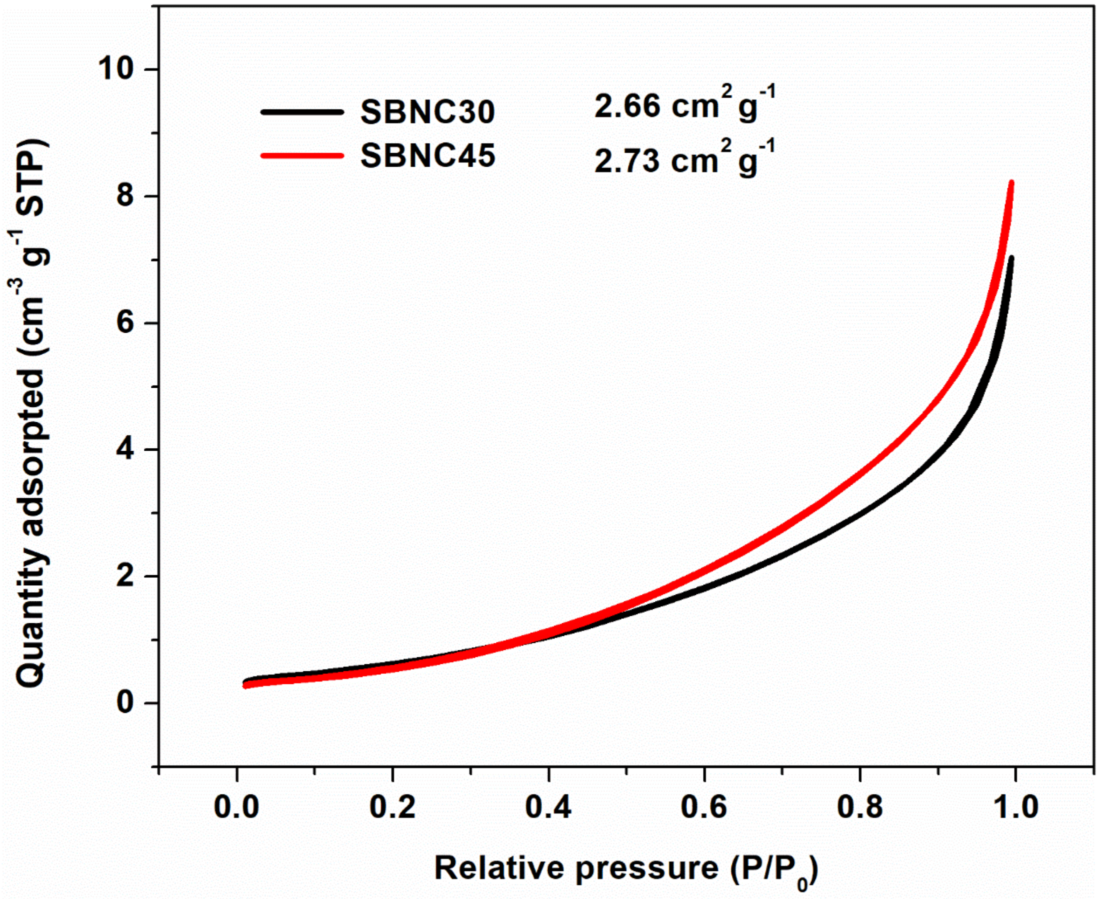

Figure S6. BET isotherms for SBNC30 and SBNC45.

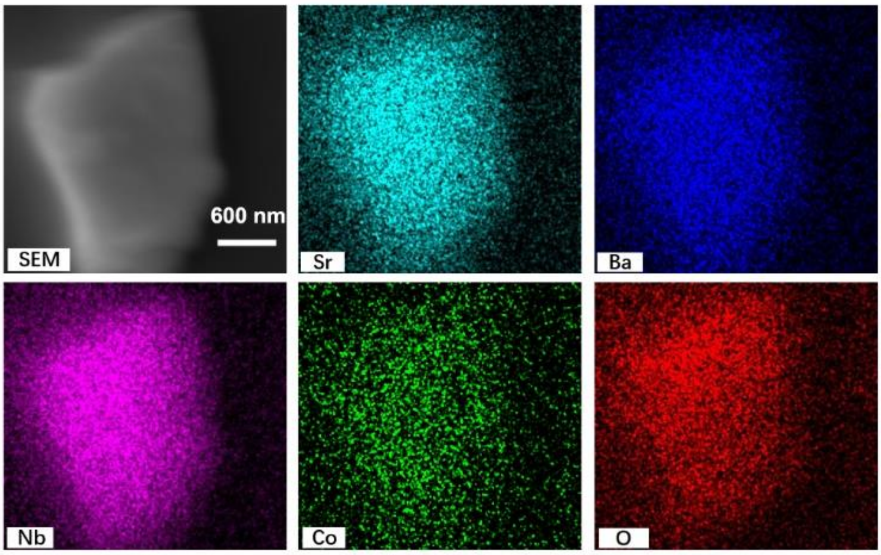

Figure S7. SEM-EDS mapping of SBNC45 sample, with the characteristic homogenous distribution of Sr, Ba, Nb, Co. and O elements on the particle.

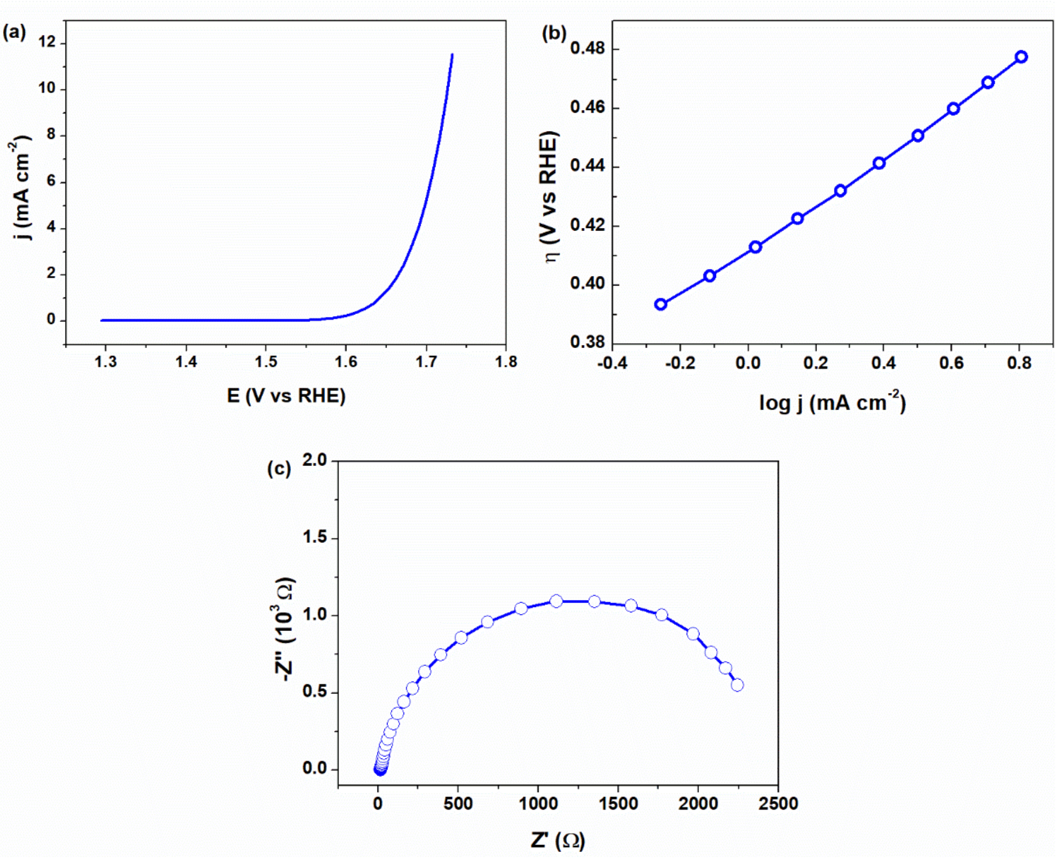

Figure S8. OER performance of KNC: (a) LSV curve with the scan rate of 5 mV s^{-1} ; (b) Tafel plot collected with the scan rate of 5 mV s^{-1}; (c) Nyquist plot measured at 1.6 V vs RHE, with the frequency ranging from 1–10^5 Hz.

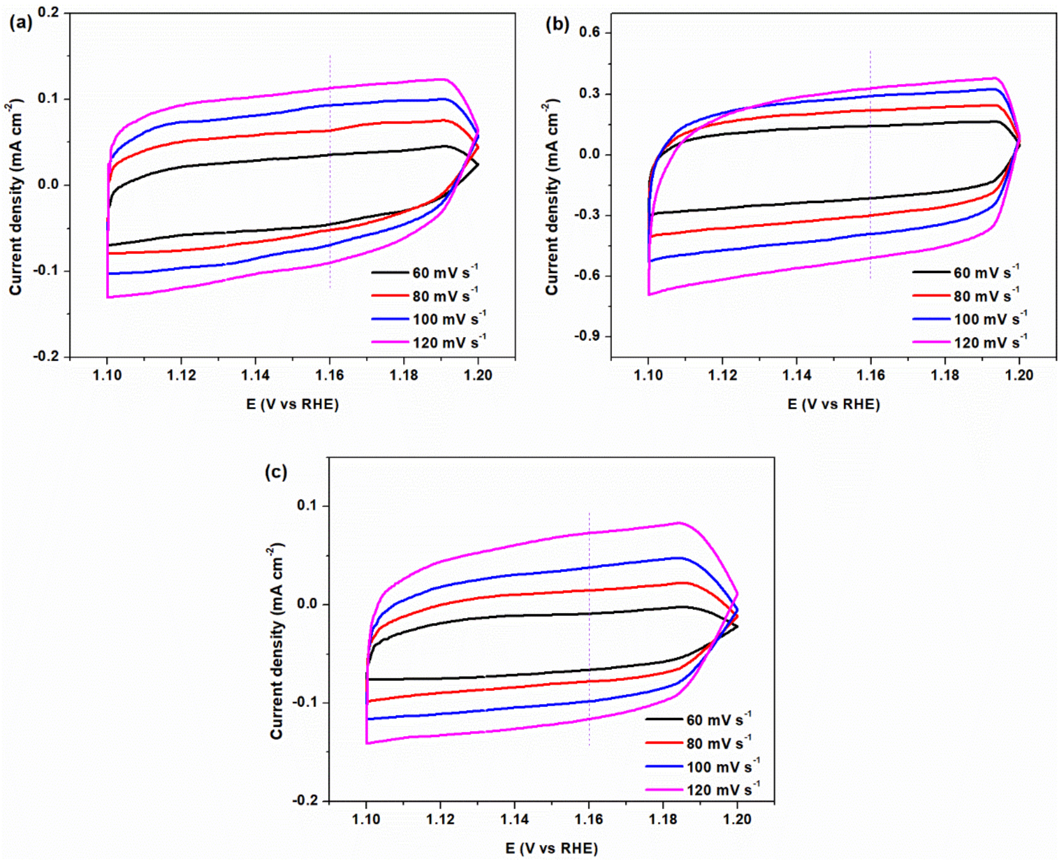

Figure S9. Cyclic voltammograms collected with different scan rates from 1.10–1.20 V vs RHE: (a) SBNC30; (b) SBNC45; (c) SBNC60.

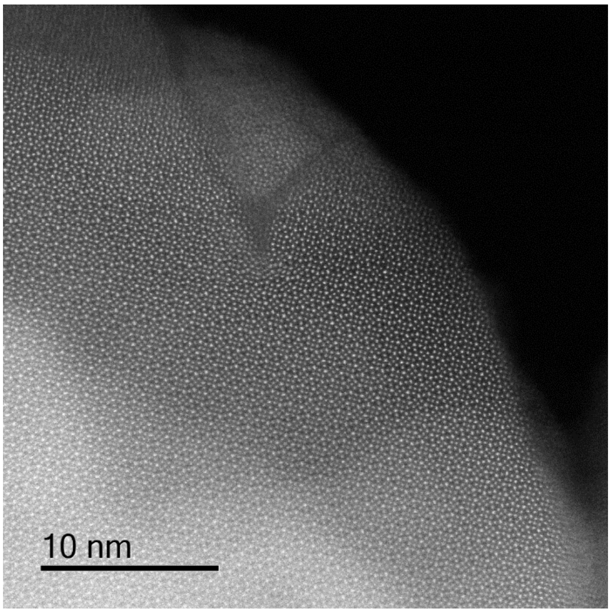

Figure S10. HRTEM-HAADF image of SBNC45 sample after the OER tests.

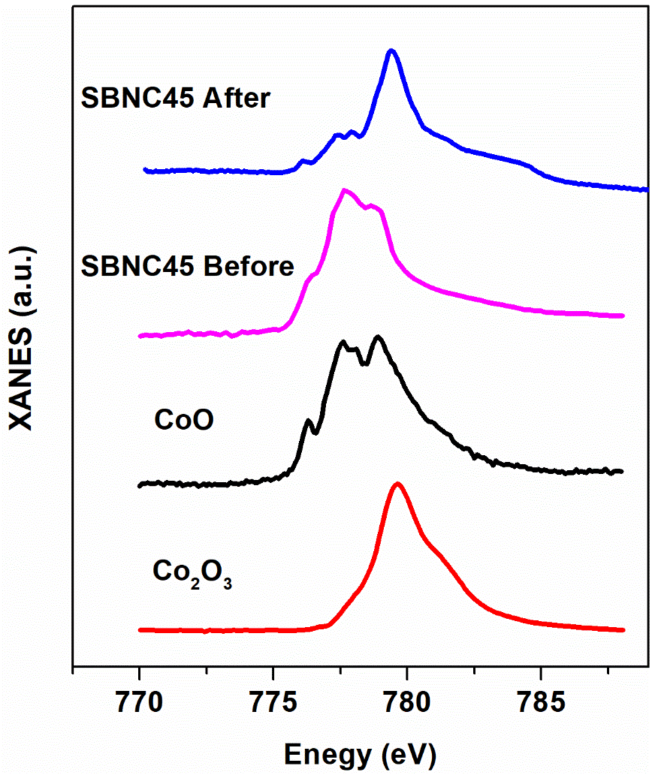

Figure S11. Co L_3-edge XAS spectra recorded on SBNC45 samples before and after the OER tests, as well as the standard reference samples.

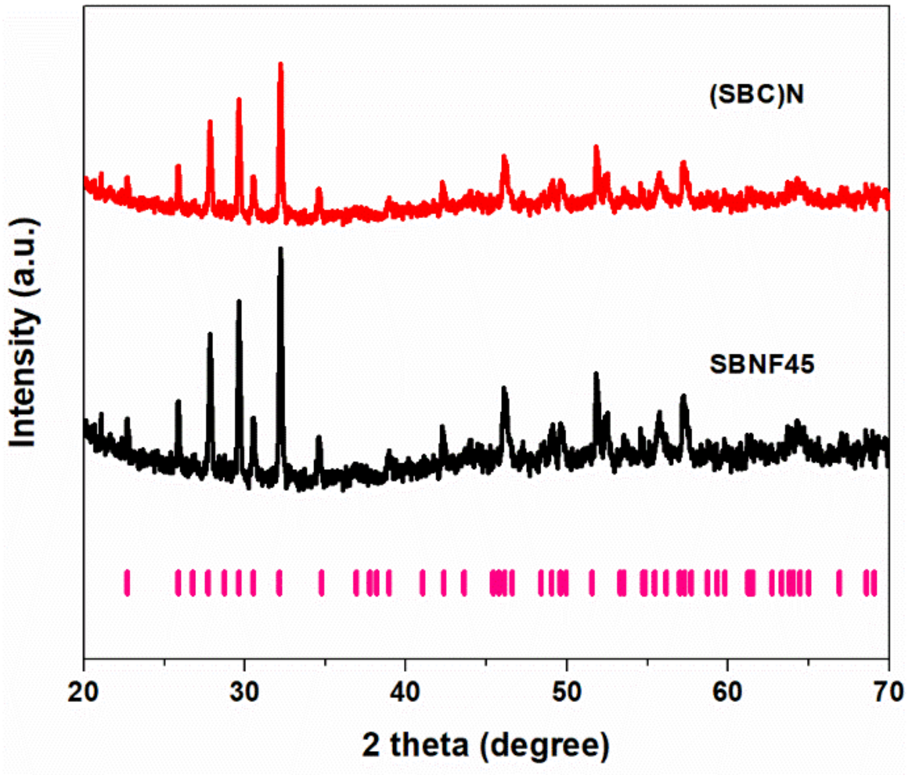

Figure S12. XRD patterns of the SBNF45 and (SBC)N samples with comparison to the standard TTB structured SBN pattern. Both SBNF45 and (SBC)N can be regarded as pure phase.

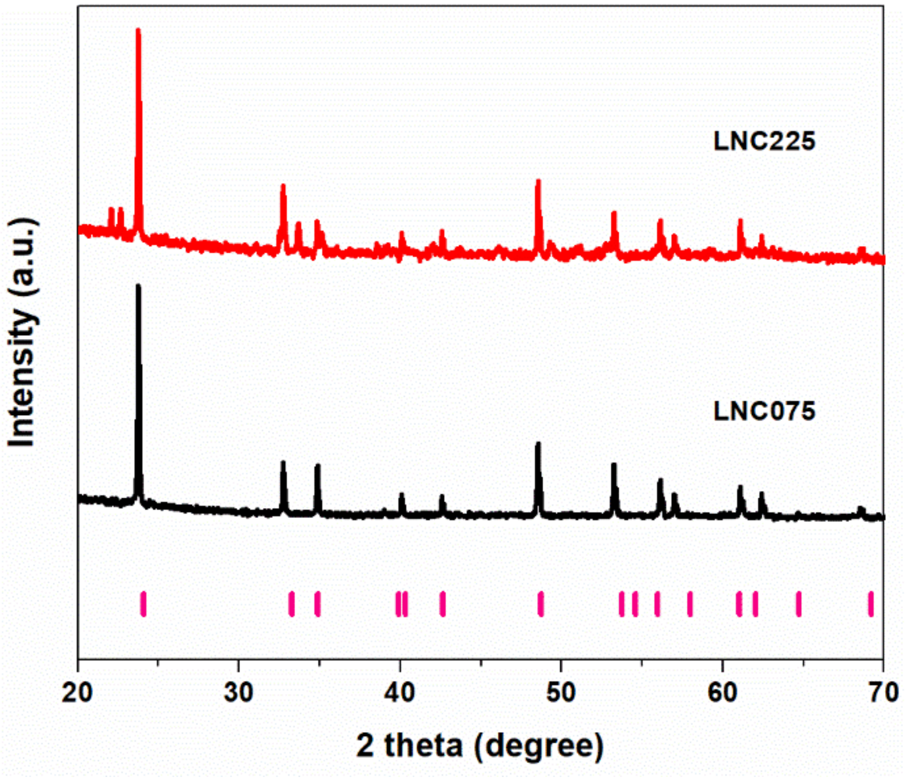

Figure S13. XRD patterns of LNC075 and LNC225 samples with comparison to the standard $LiNbO_3$ pattern. The LNC075 can be regarded as pure phase, but some impurity emerges in the LNC225 sample.

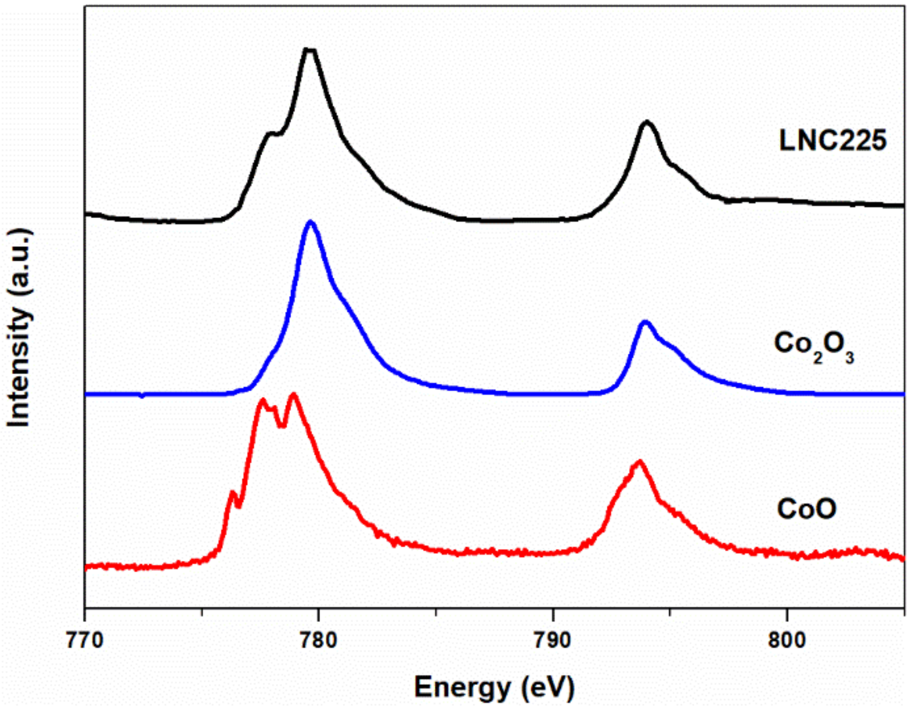

Figure S14. Co L-edge XAS spectra recorded from LNC225 and standard references CoO and Co_2O_3. Co in the LNC225 is identified as mainly 3+.

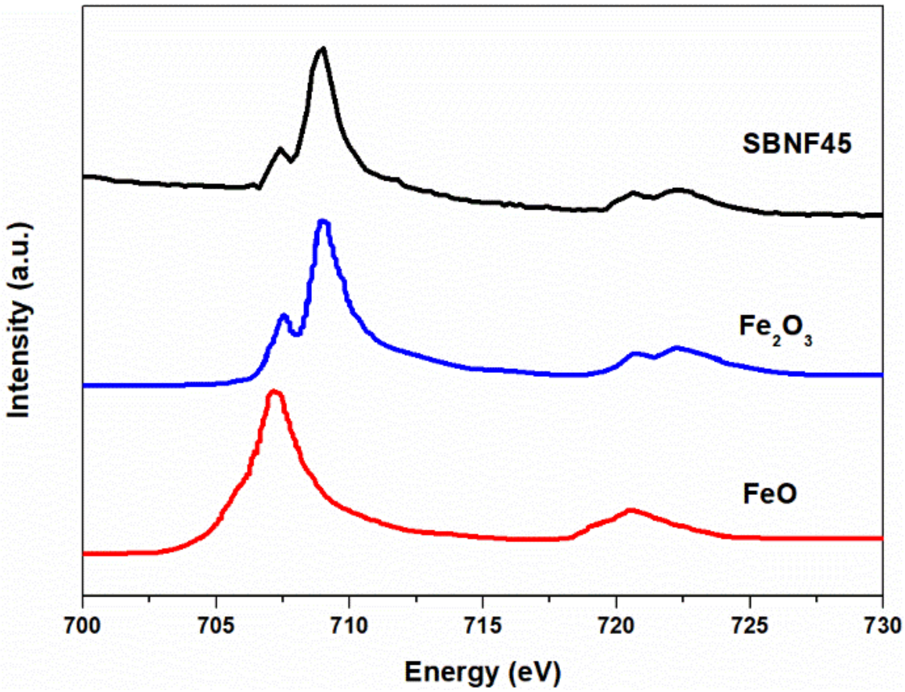

Figure S15. Fe L-edge XAS spectra recorded from SBNF45 and standard references FeO and Fe_2O_3. Fe in the SBNF45 is identified as mainly 3+.

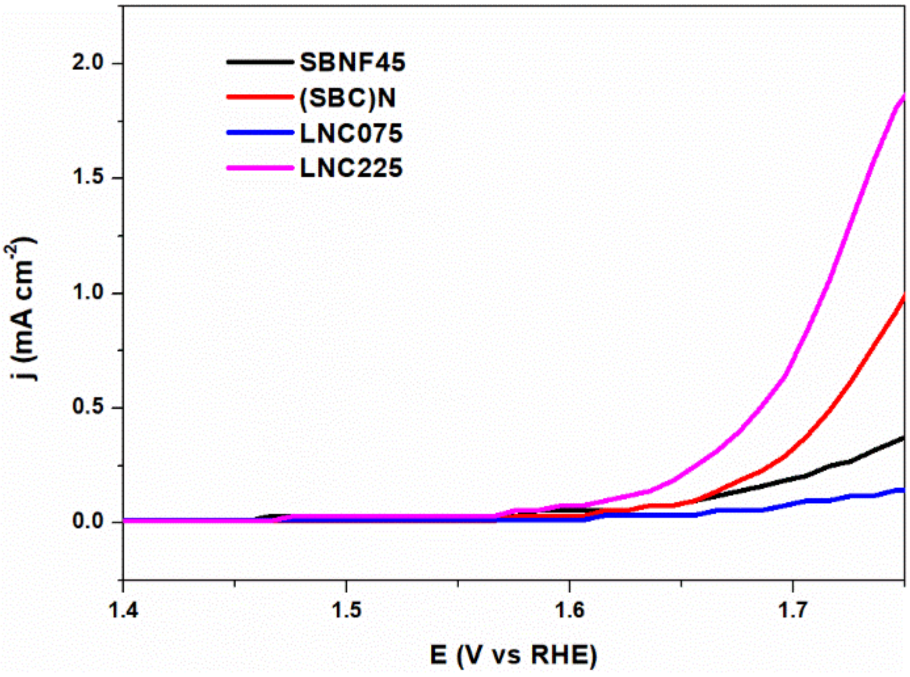

Figure S16. LSV curves of SBNF45, (SBC)N, LNC075, and LNC225 at the scan rate of 5 mV s^{-1}. These samples are not OER-active, as the current density is smaller than 1 mA cm^{-2} even at 1.7 V vs RHE.

Table S1. Structural parameters from refinement of the SBN, SBNC30, and SBNC45 samples.

SBN	x	y	z	Occ.
Nb	0.07426	0.21135	0.00129	0.720
O1	0.13908	0.06924	0.95850	0.900
O2	0.99357	0.34369	0.95880	0.750
O3	0.07540	0.20469	0.46600	0.910
Ba	0.17211	0.67211	0.49383	0.630
O4	0.28318	0.78318	0.96860	0.790
Sr	0.00000	0.00000	0.48920	0.710
Nb	0.50000	0.00000	0.01582	0.730
O5	0.50000	0.00000	0.47880	1.000
Zero-point	-0.01346	Sample displacement		0.08868
SBNC30	x	y	z	Occ.
Nb	0.07426	0.21135	0.00129	0.570
O1	0.13908	0.06924	0.95850	0.650
O2	0.99357	0.34369	0.95880	0.830
O3	0.07540	0.20469	0.46600	1.000
Ba	0.17211	0.67211	0.49383	0.470
O4	0.28318	0.78318	0.96860	0.300
Sr	0.00000	0.00000	0.48920	0.590
Nb	0.50000	0.00000	0.01582	0.530
O5	0.50000	0.00000	0.47880	0.340
Zero-point	0.02369	Sample displacement		0.03303
SBNC45	x	y	z	Occ.
Nb	0.07426	0.21135	0.00129	0.750
O1	0.13908	0.06924	0.95850	0.880
O2	0.99357	0.34369	0.95880	1.000
O3	0.07540	0.20469	0.46600	1.000
Ba	0.17211	0.67211	0.49383	0.670
O4	0.28318	0.78318	0.96860	0.820
Sr	0.00000	0.00000	0.48920	0.820
Nb	0.50000	0.00000	0.01582	0.790
O5	0.50000	0.00000	0.47880	1.000
Zero-point	0.03303	Sample displacement		0.08731

Table S2. Electronic parameters calculated based on the *M*-*T* curves and Curie-Weiss law.

	Curie constant [10^{-4} K μ_B Oe^{-1} $f.u.^{-1}$]	U_{eff} [μ_B]	Unpaired number [a] [n]	LS Co^{2+} [b] Percentage
SBNC30	1.19	4.21	3.3	Low
SBNC45	0.757	2.74	1.9	High

a) Unpaired electron number *n* has a relationship with the effective magnetic U_{eff} when total angular quantum number $L = 0$: $U_{eff}^2 = n(n+2)$

b) Oxygen vacancy also contributes unpaired electrons, which are hard to subtract; but the tendency can be roughly inferred.
